# Synthetic lethality: emerging targets and opportunities in melanoma

**DOI:** 10.1111/pcmr.12573

**Published:** 2017-03-11

**Authors:** Nicola Thompson, David J. Adams, Marco Ranzani

**Affiliations:** ^1^ Experimental Cancer GeneticsThe Wellcome Trust Sanger InstituteHinxton, CambridgeshireUK

**Keywords:** synthetic lethality, CRISPR, melanoma genomics, preclinical models, therapeutic target

## Abstract

Great progress has been made in the treatment of melanoma through use of targeted therapies and immunotherapy. One approach that has not been fully explored is synthetic lethality, which exploits somatically acquired changes, usually driver mutations, to specifically kill tumour cells. We outline the various approaches that may be applied to identify synthetic lethal interactions and define how these interactions may drive drug discovery efforts.

## Introduction

Malignant melanoma is the 5th most common cancer in the UK, with an incidence that has more than quadrupled in the last 40 years (Cancer Research UK, 2016). Melanoma originates from melanocytes, and its aetiology involves both environmental and genetic factors. Exposure to sunlight is the major environmental risk factor (Gandini et al., 2005), whilst rare germline mutations in genes such as *CDKN2A*,* POT1* and *BAP1* (Betti et al., 2016; Hussussian et al., 1994; McDonnell et al., 2016; Robles‐Espinoza et al., 2014), or common alleles such as those of the *MC1R* locus which control red hair, freckling and sun sensitivity, can also increase risk (Law et al., 2015; Robles‐Espinoza et al., 2016).

Recent advances in next‐generation sequencing have shown that cutaneous melanomas carry an extremely high mutational burden relative to other cancer types, especially in tumours arising on sun‐exposed sites (Akbani, 2015; Hodis et al., 2012). Genetic damage caused by ultraviolet radiation (UV) is associated with a distinct mutational signature, characterized by a prominence of cytidine to thymidine (C>T) substitutions as a result of erroneous nucleotide excision repair of UV‐induced pyrimidine dimers (Brash and Haseltine, 1982; Pfeifer et al., 2005). This high mutational burden makes identification of driver mutations, the lesions that have a functional role in disease initiation or progression, particularly challenging. Despite this, several important melanoma drivers have been identified including *BRAF*,* NRAS*,* NF1*,* CDKN2A* and *TP53* (Akbani, 2015; Hodis et al., 2012). Collectively mutations causing activation of the MAPK pathway are found in a large proportion of melanomas (Halaban and Krauthammer, 2016; Krauthammer et al., 2015); indeed, around 84% of cutaneous melanomas have mutations in one of the three major drivers *BRAF*,* NRAS* and *NF1* with the most frequent melanoma driver mutation being *BRAF* ^V600E^ occurring in around 40–50% of melanomas (Davies et al., 2002; Dhomen et al., 2009).

The last decade has witnessed a revolution in systemic therapy for advanced melanoma. Targeted therapies have principally been directed at the MAPK pathway as its mutation‐driven hyperactivation results in enhanced cell proliferation and consequent dependence on the pathway. The development of BRAF inhibitors targeting the BRAF^V600^ oncoprotein has significantly improved clinical outcomes, with response rates above 50% (Sosman et al., 2012). A second generation of targeted strategies has also been developed and involves combining BRAF and MEK inhibitors, thus further inhibiting MAPK signalling. This approach has further improved patient response rates to 70% and doubled the progression free survival from 6 to 12 months (Larkin et al., 2014; Robert et al., 2015a). Despite remarkable initial response rates, acquired resistance is unfortunately almost inevitable through a range of mechanisms including mutation/upregulation of the drug target, reactivation of the MAPK–ERK pathway, or hyperactivation of alternative pathways, meaning that targeting the MAPK pathway alone is rarely curative (Moriceau et al., 2015; Welsh et al., 2016).

The advent of immunotherapy has further revolutionized the treatment of advanced melanoma and has become the new standard of care. The most commonly used immunotherapies are the immune checkpoint inhibitors which block CTLA4 or PD‐1/PD‐L1, inducing the reactivation of host T cells against tumour antigens (Redman et al., 2016). The response rate of patients treated with single agent immune checkpoint inhibitors lies between 25–50%, and use of these agents has seen unprecedented long‐term disease control (Hodi et al., 2016; Robert et al., 2015b,c; Schadendorf et al., 2015). Combining CTLA4 and PD‐1/PD‐L1 inhibitors has been shown to further increase both the response rate to 61% and the overall survival to 64% at 2 years; however, these clinical benefits are associated with grade 3–4 toxicity in 36–55% of the patients (Hodi et al., 2016; Larkin et al., 2015; Postow et al., 2015). Despite representing a substantial improvement in the management of advanced melanoma, immunotherapy is currently limited by the lack of reliable biomarkers to predict which patients will respond to treatment, and a thorough understanding of the molecular mechanisms of resistance (Loo and Daud, 2016). The combination of immunotherapy and targeted therapies promises to increase the proportion of durable responders, but challenges will include the management of toxicities and adverse drug interactions (Hu‐Lieskovan et al., 2014, 2015).

Overall, although there have been substantial recent advances in the treatment of melanoma, a significant number of patients either do not respond to treatment, or respond transiently. Therefore, there is still a need to develop new therapeutic strategies.

**Figure 1:**
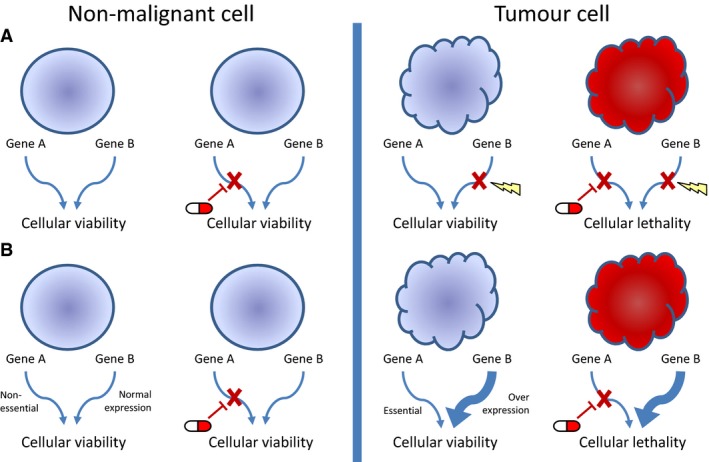
The concept of using synthetic lethality as a therapeutic strategy in cancer. (A) Synthetic lethality: The loss of gene A or gene B in isolation is compatible with cellular viability, whereas loss of both genes together leads to cellular lethality. A normal cell therefore would be able to tolerate inhibition of gene A, whilst for a tumour cell that has already lost the function of gene B, inhibition of gene A is lethal. (B) Synthetic dose lethality: Overexpression or overactivation of gene B leads to cellular dependence on gene A. Whilst normal cells are able to tolerate inhibition of gene A, in tumour cells that overexpress gene B, inhibition of gene A is lethal. Key: regular circle – non‐malignant cell; irregular circle – tumour cell; red cells are those with a complement of mutations incompatible with viability; tablet – drug inhibition; cross – loss of gene function; thickened arrow – gene over‐expression; lightning bolt – gene mutation.

Although the accrued knowledge on melanoma driver mutations has allowed their exploitation as targets for therapeutic intervention, the way these mutations interact and their role in tumour phenotype and treatment response is still not fully understood. This review will focus on synthetic lethality in melanoma, a specific genetic interaction that combines knowledge of a patient's tumour genome with defined therapeutic regimens to evoke tumour cell killing. Conceptually, synthetic lethality can be described as the scenario where loss of gene A or loss of gene B is compatible with cellular viability, whilst concurrent loss of both gene A and gene B is lethal to the cell, provoking cell death (Figure 1, top panels; Kaelin, 2005). Conversely, the term ‘synthetic dose lethality’ is used when over‐activation (rather than loss) of one gene renders another gene essential (Figure 1, bottom panels; Measday and Hieter, 2002; Megchelenbrink et al., 2015).

During malignant progression, cancers acquire multiple mutations within the genome. These mutations can have a range of effects, such as contributing towards transformation and the dysregulation of cellular pathways. Given the complexity of the genome and its functional networks, many molecular pathways/processes are characterized by redundancy: a cell can tolerate the loss of a branch of a pathway as parallel alternative pathways can exert the same function (Figure 1, left panels; Wolf et al., 2007). This redundancy, however, may expose a cancer cell carrying certain mutations to synthetic vulnerability. For example, if one pathway is no longer functional, the loss or inhibition of a second functionally parallel pathway may elicit cell death (Figure 1, right panels). In contrast, non‐transformed (normal) cells should be spared. The archetypal example of synthetic lethality is PARP inhibition in *BRCA1/2*‐deficient ovarian/breast cancer cells. In *BRCA1/2*‐deficient tumours, cells are unable to repair DNA damage through homologous repair, and subsequent inhibition of PARP, a regulator of base excision repair, leads to replication fork collapse and cell death (Farmer et al., 2005). In addition to being approved for treatment of ovarian cancer, PARP inhibitors are currently in clinical trials in a range of other tumour types with promising results (Kaufman et al., 2015; Liu et al., 2014; Mateo et al., 2015; Oza et al., 2015). In view of this, there have been ongoing efforts to find other potential synthetic lethal interactions, employing a variety of techniques to screen for further candidates. As melanomas have a high mutational burden, they may have a particularly rich landscape of synthetic vulnerabilities.

In this review, we will discuss some of the methods in use to screen for synthetic lethality, and discuss known and putative synthetic lethal targets in melanoma that may result in the development of new therapeutic approaches.

## Synthetic lethality screens

Identifying synthetically lethal gene pairs is challenging in melanoma due to the high number of mutated genes and the consequent plethora of potential gene–gene combinations. Conversely, the high mutational burden of melanoma increases the chance that a tumour may carry a mutation in a gene that is a member of a synthetic lethal (SL) pair. The identification of synthetic lethal partners in melanoma can therefore provide clinically actionable targets, which may be identified through the high‐throughput forward genetic screening approaches described in this Review. Approaches to SL screening include computational methods, drug screening, genetic manipulation with shRNA/siRNA or CRISPR, or a combination of these methods (Table 1). These techniques enable the generation and interrogation of huge amounts of data resulting in the prioritization of candidate synthetic lethal gene pairs, which subsequently require further validation, both in vitro and in vivo.

**Table 1:** Summary of common synthetic lethal screening approaches and their advantages and limitations

Synthetic lethal screening method	Advantage	Disadvantage
Yeast screens	Simple genome and easy genetic manipulation	Inability to reflect the complexity of the mammalian/cancer genome
Drug screens	Easily translated to clinical practice	Variable drug specificity Drug target sometimes unknown Limited to ‘druggable’ genes
RNAi screens	May be transcript specific Ability to target any gene within the genome Possibility of being performed in vivo	Difficult to achieve complete gene knockdown Potential toxicity of siRNA knockdown Less specific than CRISPR (off‐targets)
CRISPR screens	Possibility of achieving complete genetic knockout Ability to target both transcribed and untranscribed regions Possibility of being performed in vivo	Off‐target effects Possibility of poor guide efficiency at inducing knockout Failure of gene knockout to recapitulate drug inhibition of the target
Bioinformatic approaches	Able to utilize data from a wide range of sources, both from experiments and sequencing data	Generates long lists of potential SL pairs requiring extensive experimental validation

### Screening in yeast

The initial screens for synthetic lethality were performed in yeast, due to its small genome size and ease of genetic manipulation (Bender and Pringle, 1991; Tong et al., 2001). Some of the earliest SL screening was carried out through the mating of haploid yeast with an array of viable single mutations; the growth and fitness of the diploid double‐mutant progeny versus the non‐viable mutant combination was then assessed, to identify gene pairs that influence cellular fitness and viability (Tong and Boone, 2006; Tong et al., 2001). A high‐throughput refinement of this technique involves creation of a yeast knockout pool, with each knockout barcoded by flanking DNA sequences. A second defined mutation can then be introduced, and the relative abundance of each barcode can be read by PCR and microarray to quantify the growth of each double‐mutant strain and identify gene pairs impacting on cellular fitness and growth (Ooi et al., 2003). Several attempts have been made to map synthetic lethal gene pairs identified in yeast onto human orthologues to define candidate synthetic lethal gene partners (Chipman and Singh, 2009; Conde‐Pueyo et al., 2009; Kelley and Ideker, 2005; Li et al., 2014). This approach, however, has been challenging due to the complexity of the human versus yeast genome, and the high degree of redundancy not found in simple yeast genomes. Further, there are distinct differences in the function of gene orthologues in yeast and high‐order organisms that complicate such studies (Matuo et al., 2012; Srivas et al., 2016).

### Chemical screening to identify gene–drug synthetic lethal interactions

In human cells, a variety of screening methods can be used, including chemical screens, RNA interference and, more recently, genetic screening via clustered regularly interspaced short palindromic repeats (CRISPR) technology. Of these, chemical screens were the first to be used. These screens use a library of drugs on an array of cell lines with a specific mutational status. The effect of these drugs on cellular growth and viability is then measured. This can be performed as a high‐throughput drug screen and has been used with some success in a variety of cancer cell types (Chan et al., 2011; Iorio et al., 2016; Ji et al., 2009; Roller et al., 2012; Scortegagna et al., 2015). Iorio et al., for instance, screened across an array of cancer types, using mutation data from an extensive cell line collection combined with high‐throughput drug screening data on these lines to identify sensitizing mutations, some of which may represent SL interactions (Iorio et al., 2016).

The advantage of this approach is that drug–gene interactions can be easily identified and prioritized for preclinical and clinical validation. On the other hand, some of the compounds within the drug libraries do not have an annotated target. Additionally, drug inhibition of a target is generally less specific and effective than a genetic knockdown/knockout, due to the potential off‐target effects, and the incomplete target inhibition associated with compounds. Equally drug dose optimization to achieve the highest dose without losing specificity is not always straightforward. These complications can make downstream mechanistic studies more complex. In melanoma, high‐throughput drug screens have been performed on panels of genetically characterized cell lines; however, the high mutational burden of melanomas results in huge genetic diversity among cell lines, a factor that confounds such studies (Held et al., 2013). Drug screening has, however, identified a possible SL interaction between *PI3K/PDK1* which could be exploited to induce cell death in *PTEN* wild‐type melanoma (Scortegagna et al., 2015).

### shRNA screens for defining gene–gene synthetic lethal interactions

In contrast to chemical library screens, short‐interfering (si) and short‐hairpin (sh) RNA screens target genes at the post‐transcriptional level, through targeting and inducing degradation of specific mRNAs (Pratt and Macrae, 2009). Conceptually, this method is similar to chemical screening; however, instead of using drug compounds, RNA interference is used to screen for synthetic lethal pairs, allowing interrogation of genes and proteins which, at present, are without a specific inhibitor. A limitation of si/shRNAs (collectively known as RNAi) is the poor target specificity, with the potential for hundreds of off‐target effects (Jackson et al., 2003; Weiss et al., 2007). To mitigate this, it is common to design multiples si/shRNAs targeting the same gene to confirm phenotypes associated with a specific target (Kittler et al., 2007). It is, however, difficult to achieve complete knockdown, and in most cases, only a partial reduction in expression is observed (Boutros and Ahringer, 2008). Interpretation may be further complicated by slow protein turnover delaying the phenotypic effects of knockdown being realized, and the cellular toxicity associated with the transfection of some siRNAs may confound downstream analyses. Further, it is at present impractical to screen for the concomitant knockdown of two or more genes using si/shRNAs precluding the analysis of gene families or paralogues.

There are a variety of ways of performing si/shRNA screens, both in vitro and in vivo. Each si/shRNA can either be used in a single assay, arrayed for high‐throughput screening, or in small pools of 3‐6 si/shRNAs targeting the same transcript so‐as‐to increase knockdown efficiency. Moreover, barcoded genomewide pools of shRNAs can be delivered by lentiviral transduction into cells of interest (Berns et al., 2004; Workenhe et al., 2016). The abundance of each barcode in a pool of transduced cells can then be measured, with the relative readout (usually by DNA sequencing) representing a measure of the impact of the shRNA‐mediated gene knockdown on cell growth and survival. shRNA screens can also be performed in vivo, although it is less feasible to perform genomewide screens unless shRNAs are used in multiple pools (Possik et al., 2014). Generally, in vivo RNAi screening is performed by transducing a cellular population with an shRNA pool and then implanting this population into immunodeficient mice. At the final time point when the tumour is harvested, sequencing is performed to measure the relative representation of each shRNA compared to the original cell population, assessing the effect of gene knockdown on cellular fitness and growth (Gargiulo et al., 2014). These powerful approaches can be combined with drug treatment, and consequently, the effect of gene knockdown can be evaluated in the context of paradigms such as drug sensitivity and resistance (Yamaguchi et al., 2016). In this way, it is possible to identify synthetic lethal genes with the target of the tested compound.

A number of shRNA screens have been performed in the context of melanoma defining new synthetic lethal interactions, and mechanisms of drug resistance (Guan et al., 2015; Qin et al., 2013; Sharma et al., 2013; Smit et al., 2014). One RNAi study identified five possible SL partners with the NF*κ*B inhibitor, CDDO‐Me; although in melanoma, these SL partners are commonly overexpressed instead of being lost, thus limiting the clinical relevance of these findings (Qin et al., 2013). Another study performed a SL RNAi screen in *BRAF* ^V600E^‐mutant melanoma found that BRAF inhibition combined with ROCK1 silencing leads to cell death (Smit et al., 2014).

In addition to melanoma‐specific shRNA screens, large panels of cell lines from a diverse range of cancer types have been used to identify therapeutic vulnerabilities (Kryukov et al., 2016; Mavrakis et al., 2016). Importantly, using this approach, shRNA screens have shown that loss of expression of methylthioadenosine phosphorylase (MTAP), an enzyme involved in the methionine salvage pathway, leads to sensitization to the knockdown of PRMT5, an enzyme involved in the methylosome, in a range of malignancies including melanoma, breast and lung cancer (Kryukov et al., 2016). This phenotype has been linked to MTAP deficiency inducing the accumulation of its substrate methylthioadenosine, which inhibits PRMT5, sensitizing the cells to further PRMT5 inhibition (Kryukov et al., 2016; Mavrakis et al., 2016). Notably *MTAP* is lost in ~25% of melanomas due to its proximity to *CDKN2A*, a commonly deleted tumour suppressor gene, exposing this as a potentially therapeutically relevant vulnerability in melanoma.

### CRISPR screens to identify gene–gene synthetic lethal interactions

More recently, CRISPR technology, which enables precise and effective genomic editing, has transformed the landscape of genetic screening. This approach allows the introduction of mutations at the genetic level in a targeted manner, whereby an RNA‐guided endonuclease introduces DNA cuts. These DNA breaks are then repaired by processes such as non‐homologous end joining, introducing mutations that can result in the disruption of gene function (Shalem et al., 2014). CRISPR has the advantage that it can cause the complete knockout of a gene (or genes) of interest and has the ability to target regulatory regions such as enhancers and promoters (Xue et al., 2016). A pool of guides can be transduced into cells, and at a given time point, their relative abundance can be measured (by sequencing as described above), with depletion of a guide indicating the essentiality of a gene; in the context of a specific genetic mutation/change, a synthetic lethal interaction can be defined (Hart and Moffat, 2016; Kiessling et al., 2016). Limitations of CRISPR screens include the potential for off‐target effects, and false positives from targeting genes in highly amplified regions of the genome which may cause cell lethality independent from the effect on the target gene (Munoz et al., 2016).

At present, CRISPR screens are generally only being performed with one guide per vector, so to assess synthetic lethality, screens must be performed in a specific genetic background or in the presence of a drug/compound. Most CRISPR drug screens have been enrichment screens, looking for genes conferring drug resistance, although identification of resistance mechanisms may point to cellular dependencies and thus SL vulnerabilities (Meitinger et al., 2016; Ruiz et al., 2016; Wang et al., 2014).

In melanoma, CRISPR screens have looked for genes whose loss confers resistance to the BRAF inhibitor vemurafenib, identifying targets such as *NF1*, whose loss results in activation of *NRAS*, a recognized vemurafenib resistance mechanism (Nazarian et al., 2010; Nissan et al., 2014; Shalem et al., 2014). Screening in this manner to identify resistance mechanisms therefore has the potential to highlight signalling pathways that could be targeted via a SL approach. In addition to examining mechanisms of drug resistance, CRISPR screens may also help narrow down mechanisms of drug sensitivity at a genetic level, pointing to potential SL partners. For example, through CRISPR screening, Saha et al. showed mutation of *CDC25A* confers resistance to ATR inhibitors in embryonic stem cells. Through interrogation of biological pathways associated with this mutation, they showed that inhibition of WEE1 sensitizes cells to ATR inhibitors and induces cell death (Saha et al., 2016). Additionally, when used across a panel of cells lines with defined genetic backgrounds, CRISPR screens may point to genes essential in specific genetic contexts and possible SL partners (Hart et al., 2015; Tzelepis et al., 2016). At present, CRISPR screening is still in its infancy and emerging approaches include paired gRNAs and screens with Cas9 systems that result in gene activation instead of knockout; overall, these strategies promise to provide a great deal more information about genetic interactions and SL pairs (Konermann et al., 2015; Vidigal and Ventura, 2015; Wong et al., 2016).

### Computational approaches for identifying candidate synthetic lethal genes and pathways

In addition to in vitro techniques, computational approaches to identify synthetic lethality are able to leverage the vast amounts of genomic data available via efforts such as The Cancer Genome Atlas (TCGA) to identify putative SL gene pairs. These algorithms generally use catalogues of somatic mutations found in cancer genomes together with expression data as input and aim to identify mutual exclusivity as a marker of potential synthetic lethality. The underlying principle of such analyses is that the co‐occurrence of mutations or loss of expression of synthetic lethal gene pairs is cell lethal, and thus, they will rarely be concurrently co‐mutated or silenced. Such approaches identify large numbers of potential candidate interactions and extensive experimental validation is required (Lu et al., 2015; Srihari et al., 2015; Wappett et al., 2016).

In addition to sequencing and expression data, computational analyses can also utilize data derived from shRNA and CRISPR/Cas9 screens, particularly by amalgamating data from across multiple screens, followed by the ranking of interactions which are then validated by further experimentation (Ryan et al., 2014). A recent example of this is the Daisy approach, which utilizes data from copy number profiling, gene expression profiling and shRNA knockdown screens to identify SL pairs. This approach has been shown to identify both known and new putative SL partners, including *PARP1/BRCA1* and *MSH2/DHFR* (Jerby‐Arnon et al., 2014; Ryan et al., 2014). Of interest in melanoma, SL partners of *BRAF* ^V600E^ have also been identified by the combined use of mutation and RNAi data resulting in candidate SL interactions between *BRAF* ^V600E^ and *CXCR2*,* CDH2* and *DGKA* (Wang et al., 2016), with these interactions requiring further functional validation and mechanistic elucidation.

## Putative synthetic gene pairs in melanoma

Here, we will briefly review further knowledge of the synthetic lethal interactions that have been identified in melanoma thus far.

### DNA damage response pathways in melanoma

Cancers are inherently genomically unstable, either through the mutation of caretaker genes, responsible for DNA repair or the control of cell cycle arrest, or through oncogene induced replicative stress (Hanahan and Weinberg, 2011; Negrini et al., 2010). In the light of the clinical success of targeting the BRCA/PARP axis, the cellular response to DNA damage (DNA damage response; DDR) has received a great deal of interest as a potential SL target.

In sun‐induced melanoma, there is a high mutational burden due to the mutagenic effects of UV radiation (Hodis et al., 2012). This UV‐induced DNA damage is normally repaired by the cellular DNA repair machinery. In melanoma, however, the accumulation of C>T somatic mutations implies that these mutations have evaded repair. Although the reasons for this are currently unclear, one possible explanation is that defective cell cycle checkpoint control results in a failure of cell cycle arrest in the presence of DNA damage (Carson et al., 2012; Kaufmann et al., 2008; Pavey et al., 2013). Although *TP53* is mutated in 15% of sporadic melanoma and is a major effector of the DDR, at present there are no other known recurrent somatic mutations in genes involved in DNA repair. The importance of DNA repair in melanoma pathogenesis is, however, underscored by the 1000‐fold increase in melanoma incidence observed in patients with xeroderma pigmentosum, an inherited disorder associated with a defect in nucleotide excision DNA repair (Kraemer et al., 1994). Additionally, the importance of UV mutagenesis in the aetiology of melanoma is highlighted by the increase in melanoma mutation burden seen in individuals carrying the *R* allele of *MC1R*, resulting from a diminished ability of carriers to protect themselves from the mutagenic effects of UV damage (Robles‐Espinoza et al., 2016).

Whilst mutations in the DDR machinery are uncommon in melanoma, there are a number of putative synthetic lethal interactions that may be exploited therapeutically by specific modulation of the DNA repair machinery. One such strategy is the use of apurinic/apyrimidinic endonuclease1 (APE1) inhibitors in *PTEN*‐deficient melanomas. The *PTEN* gene codes for phosphatidylinositol‐3,4,5‐triphosphate 3‐phosphatase (PTEN), an enzyme that regulates the PI3K/AKT signalling pathway, a central pathway controlling cellular proliferation, metabolism and apoptosis, and *PTEN* has long been recognized as a tumour suppressor gene (Guldberg et al., 1997). PTEN activity is decreased in up to 65% of melanomas, both through mutation and epigenetic silencing (Aguissa‐Touré and Li, 2012; Zhou et al., 2000). Although PTEN is generally recognized for its role in the regulation of the AKT pathway, more recently PTEN has also been shown to have a role in maintaining chromosome integrity and regulating the expression of DNA repair proteins (Abbotts et al., 2014; Shen et al., 2007). *PTEN‐*deficient melanomas have decreased expression of a number of DNA repair proteins, such as RAD51 and XRCC2. Notably, further inhibition of the DDR through inhibition of APE1, a protein involved in base excision repair, causes synthetic lethality in PTEN‐deficient melanoma through increased accumulation of DNA double‐ and single‐strand breaks, which leads to apoptosis (Abbotts et al., 2014).

Additional information has been gained from in vivo SL screens, which are able to recreate some of the environmental stresses not seen in vitro. In vivo, insufficient tissue oxygenation due to the high metabolic demand and the failure of angiogenesis to keep pace with tumour growth leads to a hypoxic environment, resulting in transcriptional alterations, chromosomal instability and metabolic stress (Bedogni and Powell, 2009; Scanlon and Glazer, 2015; Vaupel and Mayer, 2007). To compare the effect of in vivo stressors on melanoma gene essentiality, a parallel RNAi screen was performed in vivo and in vitro, and genes that were uniquely selected against in the in vivo screen were further characterized (Possik et al., 2014). Of these, the DNA repair proteins ATM, CHK1 and CHK2 were selected against solely in the in vivo model, which is of interest given the impairment of the DNA damage response under hypoxic conditions (Scanlon and Glazer, 2015). Silencing of these genes was subsequently shown to significantly impair in vivo growth, and both Chk1 and Chk2 inhibitors caused significant apoptosis in vivo, with minimal effects in vitro. This effect was shown to be dependent on the hypoxic induction of HIF1*α*, as depletion of HIF1*α* in vivo protected cells against the toxicity of Chk inhibition. This is an example of a synthetic dose lethal interaction (Figure 1), with Chk1/2 inhibition being lethal in cells overexpressing HIF1*α*. This also raises the concept of synthetic lethality not only being genotype specific, but also environment specific, and that some of the unique stressors that tumours experience may lead to a genetic dependency not observed in normal tissues (Possik et al., 2014).

### *BRAF* mutation and synthetic lethality

As detailed above, the *BRAF* ^V600E^ mutation occurs in around 40–50% of melanoma, with the mutant protein having 500‐fold greater kinase activity compared to wild type (Garnett et al., 2005). The frequency of *BRAF* mutations makes it an attractive synthetic dose lethal target, due to the possible genetic dependencies consequent to hyperactivation of the MAPK pathway. To date, high‐throughput screens looking for synthetic dose lethal partners with mutant *BRAF* have identified limited synthetic lethal pairs (Wang et al., 2016). A study looking into metabolic changes in BRAF‐induced senescent cells did however find that depletion of pyruvate dehydrogenase kinase 1 (*PDK1*), a gatekeeper gene linking glycolysis to oxidative phosphorylation, selectively killed *BRAF* ^V600E^ *‐*mutant melanoma cells both in vivo and in vitro. *PDK1* was also shown to have role in mediating oncogene‐induced senescence in untransformed cells, causing cell cycle arrest upon the induction of BRAF^V600E^ expression (Kaplon et al., 2013; Scortegagna et al., 2014). PDK1 inhibition has also received attention in SL melanoma drug screens, demonstrating synergy with PI3K pathway inhibition in *PTEN* wild‐type melanoma (Scortegagna et al., 2015), and may provide a viable treatment option.

The metabolic changes induced by BRAF^V600E^ expression can also be exploited as a synthetic lethal vulnerability. Using an shRNA screen selectively targeting genes involved in metabolism in BRAF^V600E^ and BRAF wild‐type melanoma cell lines, HMG‐CoA lyase (HMGCL) was identified as a possible SL partner to BRAF^V600E^ (Kang et al., 2015). Increased levels of HMGCL, an enzyme involved in ketogenesis, were observed in BRAF^V600E^ melanomas with *HMGCL* knockdown resulting in decreased growth solely in *BRAF* ^V600E^ mutant lines. Mechanistically, it was demonstrated that the metabolite created by HMGCL, acetoacetate, enhances BRAF^V600E^ binding to MEK, and subsequent phosphorylation and activation of the MAPK pathway (Kang et al., 2015). HMGCL knockdown subsequently resulted in decreased activity of the MAPK pathway, reducing cellular proliferation and colony‐forming potential, thus pointing to a synthetic vulnerability.

### *NRAS* and *KRAS* mutations and synthetic lethality

Mutation of *NRAS* (particularly in codon Q61) occurs in around 15–20% of melanomas, with NRAS being considered an ‘undruggable target’, and thus an attractive candidate for synthetic lethal screening (Hodis et al., 2012). Although there have been no studies assessing NRAS as a synthetic dose lethal target in melanoma, in acute myeloid leukaemia (AML) the multitarget kinase inhibitor GNF‐7 has been reported to induce apoptosis in *NRAS*‐mutant but not wild‐type AML. Further mechanistic investigation using shRNA knockdown and a panel of kinase inhibitors suggest a possible synthetic dose lethal interaction between *NRAS* mutation and GCK, a serine/threonine kinase involved in the activation of JNK and MEKK1 (Nonami et al., 2015).

Although only a small subset of melanomas carry *KRAS* mutations, these mutation have received sizeable attention due their prevalence in other tumour types (Platz et al., 2008). Large‐scale screens performed in *KRAS*‐mutant lung and colorectal cancer have shown cyclin dependent kinase 1 (*CDK1*), *TBK1* and *GATA2* to be possible synthetic dose lethal partners (Barbie et al., 2009; Costa‐Cabral et al., 2016; Kumar et al., 2012), and this may be therapeutically exploitable in the small fraction of melanoma harbouring an activating *KRAS* mutation. Similarly, it was recently shown that in lung cancer *KRAS* mutations are synthetic lethal with the inhibition of the nuclear export receptor XPOI. XPOI inhibition induces nuclear accumulation of NF*κ*B inhibitors and the consequent pathway inhibition is lethal in *KRAS*‐mutant cells (Kim et al., 2016). These findings might be relevant for *KRAS*‐mutant melanoma too.

### Targeting gene paralogues to identify synthetic lethal pairs/combinations

There are a number of other approaches that may be fruitful when looking for SL pairs in melanoma. One such approach is using paralogy as a SL strategy. Paralogues are genes that have been duplicated within the genome and often have redundant roles in key physiological processes. Some of these genes may be lost as a result of passenger mutations/alternations in the cancer genome, but this is of no consequence to the cancer cells as their role is taken over by their paralogue. Targeting the paralogue of a mutated gene may therefore result in synthetic lethality if both genes are involved in an essential cellular process (Muller et al., 2015). Proof of principle has been demonstrated in glioblastoma where *ENO1,* a passenger gene frequently homozygously deleted at 1p36, leads to a synthetic vulnerability, whereby inhibition its paralogue, *ENO2*, in *ENO1‐*deficient cells is cell lethal (Muller et al., 2012).

In melanoma, the importance of targeting paralogues has been exemplified by the inhibition of the MAPK kinase pathway through the use of MEK inhibitors. MEK has two functionally redundant paralogues: MEK1 and MEK2 (Aoidi et al., 2016). In an in vivo model of *BRAF* ^*V600E*^‐mutant melanoma, the combination of BRAF inhibition with either MEK1 or MEK2 knockdown resulted in a modest reduction in metastasis. In contrast, combined BRAF inhibition and knockout of both MEK1 and MEK2 resulted in a dramatic reduction in metastasis, demonstrating the importance of targeting functional redundancy within signalling pathways (Sharma et al., 2006). The MEK inhibitors currently in use clinically target both MEK1 and MEK2 and when combined with BRAF inhibitors in patients with *BRAF* ^V600E^‐mutant melanoma prolong progression free survival by an average of 4 months in metastatic disease (Robert et al., 2015a). Other therapeutic strategies involving targeting of kinase pathways are likely to require inhibition of multiple isoforms, with key kinases such as ERK and AKT existing as multiple paralogues (Cohen, 2013; Roskoski, 2012).

### *MYC* and synthetic lethality

Another putative therapeutic strategy is targeting *MYC* overexpression, as a synthetic dose lethal strategy. *MYC* is a proto‐oncogene and transcription factor that is amplified or overexpressed in around 6% of melanomas (Akbani, 2015). A pooled genomewide shRNA screen in mammary cancer cell lines identified *BUD31*, a gene involved in spliceosome catalytic activity, as a synthetic dose lethal partner of *MYC* amplification (Hsu et al., 2015). This may be due to the increased amount of total cellular mRNA generated through *MYC* over‐expression exerting pressure on the splicing machinery leading to the induction of cell death in the absence of BUD31. Of note, *MYC* upregulation has different effects on the spliceosome in different tumour types and it remains to be seen if this effect carries over into melanoma (Koh et al., 2015). Another synthetic dose lethal partner of *MYC*, identified through shRNA screening, is *MAP3K13* which is a kinase involved in MYC phosphorylation, a process that maintains MYC stability and transcriptional activity (Han et al., 2016). In *MYC* overexpressing breast cancer cell lines, inhibition of MAP3K13 leads to impaired colony formation in vitro and tumour regression in vivo, warranting further investigation of this synthetic dose lethal pair in melanoma.

## Conclusions and perspective

Given its high mutational burden, melanoma is likely to carry genetic lesions that confer molecular vulnerabilities. The study of synthetic lethality may identify new therapeutic targets, enabling the development of new and effective treatment regimens. Moreover, identification of new SL pairs has the potential to drive drug discovery and enable rational testing of drug combinations with huge clinical translational potential. Clinical translation is likely to require collaboration with drug discovery units in order to identify novel specific inhibitors.

Current areas of interest include exploiting the DNA damage response, metabolic reprogramming and aberrant receptor tyrosine kinase signalling pathways. At present, relatively few SL pairs have been identified and conclusively validated in melanoma; however there are ongoing efforts to redress this issue. Further, advances in genome engineering through CRISPR/Cas9 technology will provide an effective platform on which to perform large‐scale screens to identify new synthetic lethal partners, and build upon existing knowledge about gene pair interactions. Future discoveries in synthetic lethality promise to unveil new therapeutic avenues for effective and personalized treatment of melanoma.

## Funding

NT is supported by a Wellcome Trust Sanger Institute Clinical PhD studentship. DJA and MR are supported by Cancer Research UK, The Wellcome Trust (WT098051) and the ERC Synergy Programme (Combat Cancer).

## Conflict of interests

The authors declare no conflict of interests.
